# Louse Flies on the Fly: Host Macroecology Shapes Interspecific Variation in Ectoparasite Prevalence Among Migrating Birds

**DOI:** 10.1002/ece3.73846

**Published:** 2026-06-18

**Authors:** Aleksandra Janiszewska, Piotr Minias, Radosław Włodarczyk, Maciej Kamiński, Dariusz Jakubas, Magdalena Remisiewicz, Hanna Sztwiertnia, Maciej Bartos

**Affiliations:** ^1^ Faculty of Biology and Environmental Protection, Department of Biodiversity Studies and Bioeducation University of Lodz Lodz Poland; ^2^ Faculty of Biology, Department of Vertebrate Ecology and Zoology University of Gdansk Gdansk Poland; ^3^ Faculty of Biology, Bird Migration Research Station University of Gdańsk Gdansk Poland; ^4^ Silesian Ornithological Society Wroclaw Poland

**Keywords:** birds, comparative analysis, ectoparasites, host–parasite interactions, louse flies, *Ornithomya*

## Abstract

The prevalence of ectoparasites is strongly affected by the host environment, which shapes not only host exposure, but also parasite transmission and survival. Louse flies (Diptera: Hippoboscidae) are obligate blood‐feeding ectoparasites of birds and mammals, showing morphological adaptations to permanent life on the host body, such as dorsoventrally flattened bodies and strong claws. Here, we conducted a broad‐scale screening of louse fly prevalence across 157 avian host species captured during autumn migration through Central Europe (over 100,000 individuals screened). We also ran a phylogenetically informed comparative analysis to identify host traits shaping interspecific variation in louse fly prevalence. We identified seven louse fly species across the entire sample, including two dominant species (*Ornithomya avicularia* and *O. fringillina*). The median louse fly prevalence across all host species was low (median prevalence 2.2% infected host individuals; 67% infected host species). Phylogenetically‐informed Bayesian models revealed that the total louse fly prevalence was significantly associated with host species, body mass, precipitation within the host breeding range, host habitat type, trophic niche and migration distance. However, models run separately for the two most prevalent louse fly species (*O. avicularia* and *O. fringillina*) showed contrasting (species‐specific) effects of host traits. Our results revealed that interspecific variation in the prevalence of the obligate avian ectoparasites is shaped by a complex combination of host life‐history traits, habitat features and climatic conditions. The study provides novel insights into the ecological and environmental determinants of host–parasite associations, shedding light on how biodiversity patterns of parasites and their hosts are shaped across species and habitats. Because climate‐driven shifts in migration strategies and environmental conditions are likely to reshape host–parasite interactions, identifying host traits associated with susceptibility to ectoparasites is essential for predicting parasite risks and informing conservation.

## Graphical abstract

We analysed over 100,000 migrating birds from 157 species to identify macroecological drivers of interspecific variation in louse fly prevalence. Using phylogenetically informed Bayesian models, we show that host morphology, ecology and biogeographic traits shape infection patterns, with *Ornithomya avicularia* and *O. fringillina* exhibiting contrasting associations. Our findings highlight how broad‐scale variation in host traits and migratory strategies influence parasite dynamics across species.

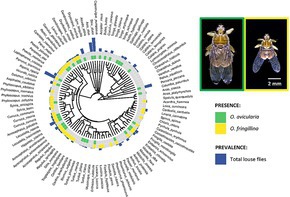

## 1 Introduction

Host–parasite relationships represent some of the most intricate forms of ecological interactions in the natural world. Parasites strive to optimise their transmission and survival, while hosts evolve defences to avoid exploitation (Dawkins and Krebs 1979). This ongoing coevolutionary arms race is widely recognised as the major driver of biological diversity (Hall et al. 2011). In the early evolutionary stages of parasitism, parasites often exhibit low host specificity and limited anatomical or physiological adaptations to particular hosts (Boeger et al. 2003). However, over evolutionary time, close host–parasite associations tend to promote the development of highly specialised parasitic strategies and traits that enhance exploitation of fewer and less diverse hosts (Brockhurst et al. 2014). This process of parasitic specialisation, shaped by host ecology, behaviour and immune responses, is thought to be favoured over generalist strategies, primarily due to the higher physiological and ecological costs of the latter (Fain 1994; Vázquez et al. 2005). Parasite prevalence is among the key parameters for understanding the ecological dimension of host–parasite interactions. It is governed by a range of host‐related behavioural, environmental and demographic factors, and can serve as an indicator of host population health or environmental disturbance (Mbora and McPeek 2009; Turcotte et al. 2018; Kołodziej‐Sobocińska 2019). Comparative analyses of parasite prevalence among host species are traditionally used to investigate variation in host susceptibility, as well as identify species that may serve as key reservoirs or transmission hubs.

Ectoparasites have evolved a variety of adaptations to take advantage of their hosts and survive in a specific external environment, such as the body surface of their hosts (Marshall 1981; Poulin 2007). In contrast to internal parasites, ectoparasites are continuously exposed not only to environmental factors, but also to host defensive activities, such as grooming, preening, sand bathing or skin secretions. Hence, many ectoparasites have developed morphological, physiological and behavioural counter‐adaptations that closely match host biology (Clayton and Moore 2013; Price et al. 2003). These include the development of specialised claws for adhering to host integument, flattened bodies for travelling through feathers or fur, and cryptic colouration or microhabitat preferences that make them more difficult to locate and identify by the host (Bush et al. 2010; Johnson et al. 2012; Abad‐Franch et al. 2021).

Ectoparasite prevalence is also significantly influenced by the host environment. For instance, the level of ectoparasite activity is often determined by ambient temperature (Franke et al. 2017; Holand et al. 2019) and humidity (Moyer et al. 2002; Dube et al. 2018). The effects of environmental factors on ectoparasite activity and prevalence may also be modulated by climate changes (Heath 2020; Ajith et al. 2020), as well as anthropogenic degradation of host habitats (Shilereyo et al. 2022; Babyesiza et al. 2023; Gebrezgiher et al. 2023).

Louse flies (Diptera: Hippoboscidae) are hematophagous ectoparasites of warm‐blooded animals. So far, 213 louse fly species have been described worldwide, most of which parasitise birds. A characteristic feature of this group is adenotrophic viviparity, a remarkable reproductive strategy in which larval development takes place in the female's body. After being deposited, the fully developed larva immediately pupates, forming a specific type of puparium. Transmission of avian louse flies most often occurs in the host nest, where puparia typically overwinter. Following metamorphosis in spring, adult flies emerge and infest both adult birds and their nestlings (Hutson 1984). Compared to other dipterans, adult louse flies are relatively long‐lived animals. Data available for a few species indicate that adults may survive from several weeks to a few months (Hutson 1984; Lehane 2005). However, accurate estimates of lifespan remain scarce for most Hippoboscidae, due to their strong association with hosts and the difficulty of maintaining them under laboratory conditions. Many louse fly species exhibit varying degrees of host specificity. For example, *Ornithomya avicularia* is one of the most common louse flies parasitising a variety of bird species (both passerines and non‐passerines) across the Palearctic region (Oboňa et al. 2019), whereas *Ornithomya fringillina* mainly targets passerine birds, although it is also less frequently found on species from other avian orders. It has been recognised that biological characteristics of birds, such as habitat type, migration strategy, nesting method or lifestyle (e.g., colonial vs. solitary), can affect infestation risk and the intensity of host colonisation by louse flies (Santolíková et al. 2022; Keve et al. 2024). However, rigorous comparative analyses of louse fly prevalence across a broad spectrum of phylogenetically diverse host species are virtually lacking. Among the exceptions, a comparative analysis of louse fly prevalence estimates from nearly 10,000 bird individuals of 134 species (Finland) provided clear evidence for the effects of host breeding habitat and nesting strategy on infestation by these ectoparasites (Lehikoinen et al. 2021). The data used in these analyses originated, in significant part, from breeding birds, which could promote associations of louse fly prevalence with host breeding biology. It may though be expected that different sets of host traits could better predict interspecific variation in louse fly prevalence during nonbreeding stages of host annual cycle, such as seasonal migration.

The aim of our study was to investigate variation in louse fly prevalence among avian host species during autumn migration. For this purpose, we quantified louse fly prevalence across over 100,000 individuals of 157 species migrating through Central Europe (Poland). First, we tested for the associations of the total louse fly prevalence with a broad spectrum of host traits related to ecology, biogeography, behaviour, phenology and population age structure. Second, we collected over 1000 louse fly specimens for species identification and investigated the patterns of interspecific variation in the prevalence of *O. avicularia* and *O. fringillina*—two most common louse fly species parasitising birds in Europe. We expected that the prevalence of both louse fly species may be shaped by different sets of host traits, due to differences in the degree of their host specificity and host preferences.

## 2 Materials and Methods

### 2.1 Study Sites and Data Collection

**FIGURE 1:**
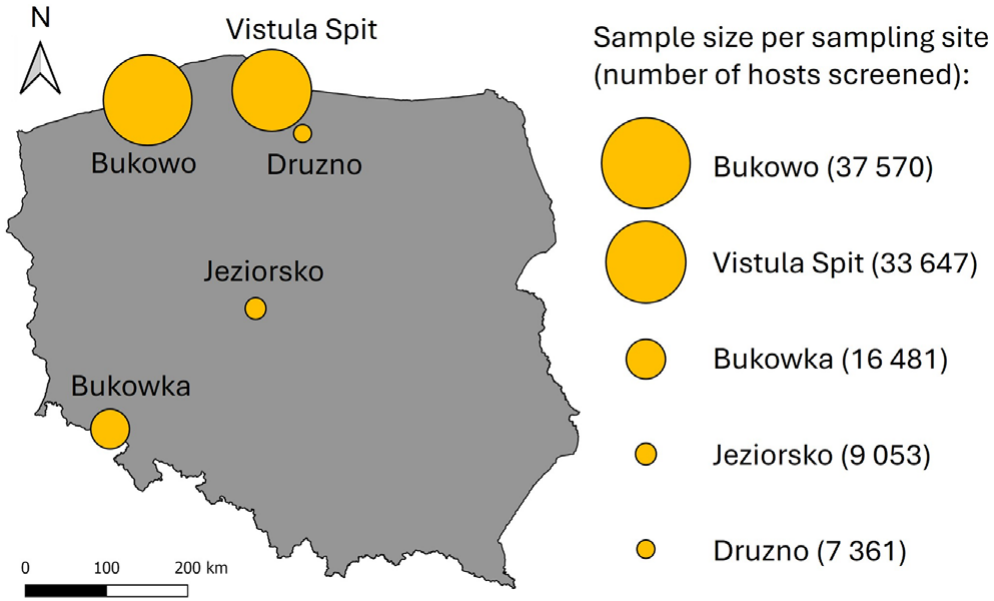
Map of Poland with sampling sites. The number of checked bird individuals at each location is represented by point size; the larger the point, the higher the numbers of checked bird individuals.

The data on louse fly prevalence were collected between 2014 and 2023 during the autumn bird migration period (late July to mid‐November). Fieldwork was conducted at four sites scattered across Poland: at the southern Baltic coast (stations: Mierzeja Wiślana at the Vistula Spit and Bukowo, at the spit between Lake Bukowo and the Baltic Sea), in the Żuławy Alluvial Plain in northern Poland (Druzno Lake), central lowlands (Jeziorsko Reservoir) and uplands of the central Sudetes Mountains in southern Poland (Bukówka Reservoir) (Figure 1). Birds were captured as part of long‐term bird ringing programs using mist nets and funnel traps. Each captured bird was weighed, measured and aged according to standard bird ringing protocols. Afterwards, wing and body feathers were carefully inspected for the presence of louse flies. Louse flies noticed on the earlier stages of bird handling (in the mist nests or during transport/ringing) were also recorded. Bird age was categorised into two classes based on plumage (Demongin 2016): juvenile or adult, with juveniles representing the large majority of all inspected individuals (87.2%). For each species, the median capture date was calculated as a phenological index of peak migration (ranging from 30th of July to 2nd of November).

In total, *n* = 104,112 individuals from 157 bird species were inspected for the occurrence of louse flies, representing 15 avian orders, 41 families and 79 genera. The data were poorly balanced in terms of host taxonomic composition and sample sizes, as most species (*n* = 93) originated from a single order of Passeriformes and sample sizes ranged from a single screened individual to several thousand individuals (max. 17,302 individuals for the European robin *Erithacus rubecula* ) per species (median = 33 individuals). The prevalence of louse flies was calculated as the proportion of the number of infected individuals to the total number of inspected individuals per each host species. The prevalence was calculated and reported for 105 host species (*n* = 102,748 individuals) with a sample size of at least 10 inspected individuals (representing 10 avian orders and 31 families). The remaining 52 species had a sample size of < 10 inspected individuals. When technically possible, each bird was handled individually and kept in a separate cloth bag, minimising the risk of ectoparasite transfer between individuals. Occasionally, conspecific birds were temporarily kept in a single container, which still minimised the risk of ectoparasite transfer between host species.

A proportion (67.4%) of all detected louse flies was collected (manually or with forceps) and preserved in 96% ethanol. All collected specimens were subsequently identified under a stereomicroscope following the taxonomic keys developed by Borowiec (1984) and Hutson (1984). The morphological identification of randomly selected specimens from each species was validated by molecular barcoding approach (protocols described in Rewicz et al. 2021). It was technically unfeasible to collect all detected louse flies, as some individuals escaped during host handling. All louse fly species are highly mobile and may rapidly abandon the host when disturbed, which can hinder effective sampling. However, our sampling procedures were highly consistent across host species, sites and years; therefore, the observed species composition was expected to be well representative of the louse fly community. The prevalence of each louse fly species was quantified in two ways: (i) raw counts of identified specimens (without adjustment for unsampled specimens), and (ii) identification‐adjusted counts, obtained by extrapolating observed species composition to the total number of detected louse flies, including unsampled (unidentified) specimens.

### 2.2 Host Traits

In order to identify macroecological drivers shaping interspecific variation in the louse fly prevalence across bird species, we quantified various biogeographical, climatic, ecological and life‐history traits of avian hosts. First, to quantify mean breeding latitude and migration distance (km), we used breeding and nonbreeding distributional range maps obtained from BirdLife International and Handbook of the Birds of the World (2016). Following the methodology described in Minias and Włodarczyk (2020), we calculated the geographic centroid of breeding and wintering range using the *gCentroid* function from the *rgeos* package (Bivand and Rundel 2023) developed for the R statistical environment (R Foundation for Statistical Computing, Vienna, Austria). Breeding latitude was expressed as the latitude of the centroid of the breeding range (breeding/resident spatial polygons). Migration distance was calculated as the distance between the centroids of breeding and nonbreeding (nonbreeding/resident spatial polygons) ranges.

To quantify climatic conditions within the breeding ranges of host species, we extracted spatial data on annual mean temperature (°C) and annual mean precipitation (mm). Information on both climatic variables was obtained from the WorldClim database v2.0 (Fick and Hijmans 2017) at a spatial resolution of 10 arc‐minutes. Using breeding and resident range polygons (BirdLife International), we calculated the average value of each climatic variable across the entire breeding range of each species. Calculations were performed in R software using the *lets.summarizer* function from the *letsR* package (Vilela and Villalobos 2015).

Next, we used published datasets to compile information on three ecological and reproductive host traits, including trophic niche (AVONET database; Tobias et al. 2022), as well as habitat type and nest placement (Tobias and Pigot 2019). Trophic niche was grouped into five categories: aquatic predator (30 species), herbivore (20 species), invertivore (74 species), omnivore (21 species), vertivore (12 species). Habitat type was grouped into four major categories: woodland (including forest; 69 species), wetland (including riverine; 44 species), grassland (16 species) and shrubland (14 species). The remaining habitats with low representation (< 10 species) in the data set (i.e., coastal, rock and human‐modified habitats) were grouped into a single category (henceforth referred to as other habitats; 14 species). Nest placement was classified in a simple three‐way system as cavity (52 species), exposed elevated (57 species) and exposed ground nest (48 species).

Finally, information on body mass (g) and clutch size (number of eggs) was compiled from monographies of birds of the world (del Hoyo et al. 1992–2011; Snow and Perrins 1998), with additional body mass data extracted from Dunning (2008). Due to right‐skewed distributions, both body mass and clutch size were log‐transformed prior to statistical analyses.

### 2.3 Comparative Analyses

To investigate variation in louse fly prevalence across avian species, we employed Bayesian phylogenetically informed mixed models (Hadfield and Nakagawa 2010) implemented in the *MCMCglmm* R package (Hadfield 2010). First, the number of host individuals (birds) infected with louse flies (regardless of species) was used as the response variable and modelled as count data using a Poisson distribution. Second, we ran similar models using the number of birds infected with either of the two most prevalent louse fly species (*O. avicularia* and *O. fringillina*) as the response variables (raw prevalence). Predictors (fixed effects and covariates) included biogeographic (host species breeding latitude and migration distance), climatic (annual mean temperature and annual mean precipitation within host breeding range), ecological (habitat type, trophic niche and nest placement) and life history (body mass and clutch size) traits. We also included host age structure (proportion of adults) and median capture date as additional predictors. All these models were run with species as the unit of analysis. However, to examine temporal variation in louse fly prevalence, the data were recoded at the year level. In this analysis, total louse fly prevalence (per species per year) was used as the response variable, while year was included either as a linear covariate (to test for directional changes in prevalence over the sampling period) or as a fixed factor (to test for differences in prevalence among years).

**FIGURE 2:**
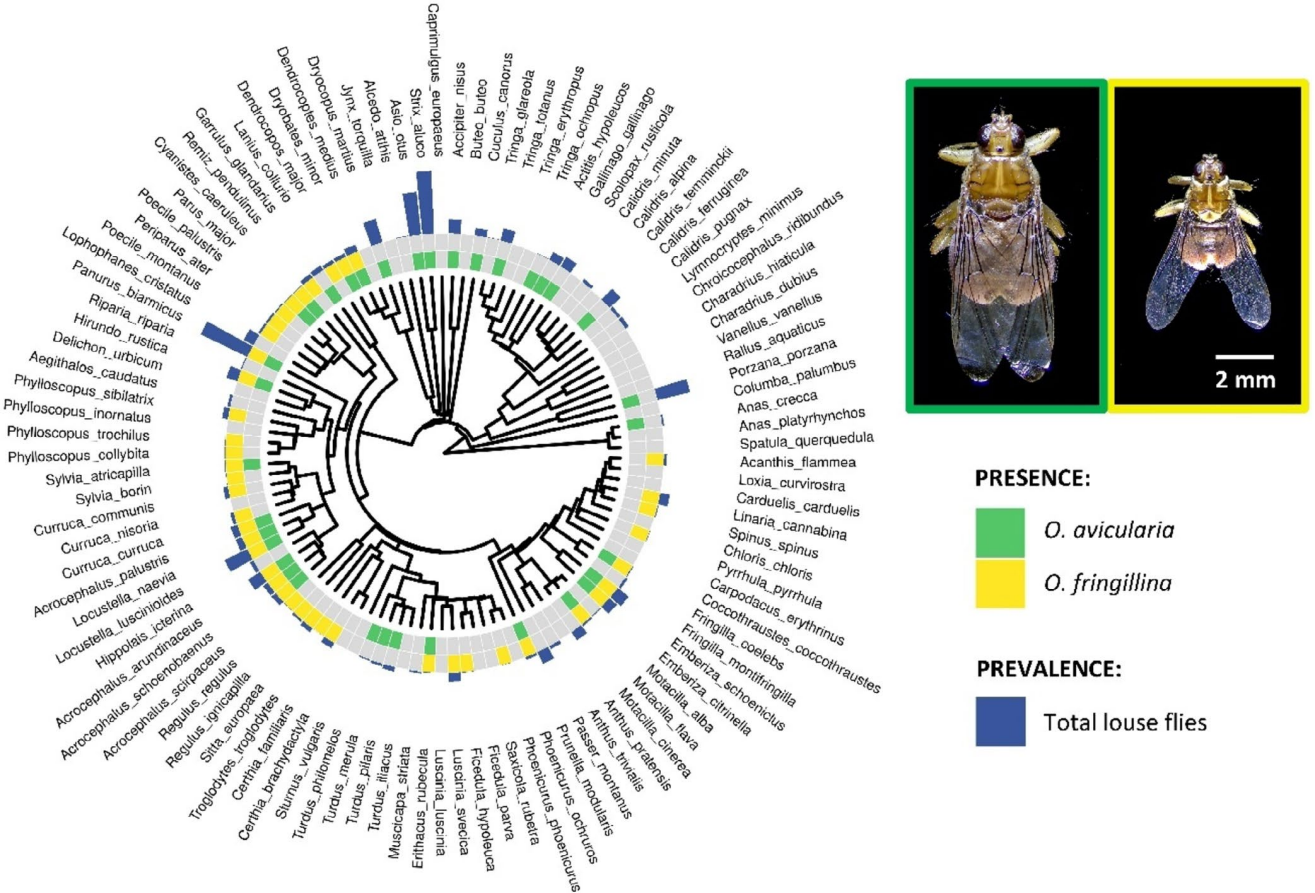
Distribution of louse fly taxa across avian hosts. Green bars—*Ornithomya avicularia*, yellow bars—*O. fringillina*, blue bars—all louse fly species combined. Both photos were taken at the same magnification.

The total sample size (number of host individuals screened for louse fly presence) was included as an offset in each model. To account for phylogenetic nonindependence among host species, we included a random effect of species identity linked to phylogeny. Phylogenetic relationships were reconstructed based on the comprehensive time‐calibrated avian tree available at the BirdTree webserver (Jetz et al. 2021). To address phylogenetic uncertainty, we first retrieved 1000 alternative trees for our study host species using a backbone topology described by Ericson et al. (2006) and then used Geneious v.10.0.5 (Biomatters Ltd., Auckland, New Zealand) to summarise all alternative trees into a single consensus tree that was used in the analyses (Figure 2).

Uninformative priors were specified for the variance components (variance set to one and belief parameter set to 0.002 in R and G structures), but a strong prior was used to hold the coefficient associated with offset at one (B structure). Markov chain Monte Carlo simulations were run for 500,000 iterations, with a burn‐in of 100,000 and a thinning interval of 400, yielding 1000 expected posterior samples per chain. Consistent with this expectation, an average effective sample size for model parameters was 948.7 ± 129.3 [SE]. Chain convergence was assessed using two alternative approaches: (i) inspection of trace plots (Roy 2020) and (ii) application of Geweke diagnostic, that is, *z*‐score tests investigating the difference in the means of the first (10%) and the last part (50%) of each chain (*z* < 1.96 was used to infer sufficient convergence) (Geweke 1992).

Originally, all the models were run on the full dataset (*n* = 157 bird species). However, to check if our results were not biased by the presence of host species with small sample sizes (possibly yielding inaccurate estimates of louse fly prevalence), we subsampled the data based on the sample size threshold (*n* = 105 bird species with at least 10 screened individuals) and re‐ran all the models. Also, to assess whether the results for the two most prevalent louse fly species were influenced by the presence of unsampled/unidentified specimens, we re‐ran these models (full data set) using identification‐adjusted prevalence as the response variable. To obtain more parsimonious reduced models, highly nonsignificant predictors (*p* > 0.25) were removed from the full models. Model reduction was conducted in a single step to avoid biases (inflation of Type I error rates) caused by multiple testing under the traditional stepwise approach (Mundry and Nunn 2008). All values are reported as means ± SE.

## 3 Results

### 3.1 Louse Fly Prevalence and Host‐Specificity

Approximately half of all studied bird species (79 out of 157) were found to be infected with louse flies. However, after excluding species with low sample sizes (< 10 individuals screened), the proportion of infected bird species increased to ca. 70% (73 out of 105; Figure 2). Among infected species, the total louse fly prevalence ranged from 0.07% to 26.7%, while median prevalence was 2.2% (Figure 2). Exceptionally high prevalences of louse flies were recorded in some nonpasserine bird species, especially owls (Strigidae): tawny owl (*Strix aluco*)—26.7% and long‐eared owl (*Asio otus*)—18.2%, but also in passerine species, for example, the bearded reedling (*Panurus biarmicus*)—22.1% (Figure 2).

Seven species of louse flies from three genera were identified across all host species: *O. fringillina, O. avicularia, O. biloba, O. chloropus*, *Icosta ardeae*, *Lipoptena fortisetosa* and *L. cervi* . Two species (*O. fringillina* and *O. avicularia*) were found to be clearly dominant in our dataset (49.0% and 36.8% of all collected louse flies, respectively). *O. fringillina* showed a relatively high degree of host specificity and was mostly associated with small passerines (recorded in 40 passerine species), with only two specimens recorded in nonpasserines (woodpeckers Picidae) (Figure 2). It was particularly prevalent in wetland‐associated passerines, such as the bearded reedling (11.4%), Savi's warbler *Locustella luscinioides* (4.2%) and bluethroat *Luscinia svecica* (3.8%). In contrast, *O. avicularia* was more generalist, being recorded in 40 phylogenetically diverse host species representing eight avian orders (Figure 2). It was most prevalent in divergent nonpasserine hosts, such as the tawny owl (20%), long‐eared owl (18.6%), black woodpecker *Dryocopus martius* (7.7%), and spotted crake *Porzana porzana* (7.1%). The remaining two *Ornithomya* species ( *O. biloba* and *O. chloropus*) were much less common (8.7% and 3.9% of all collected louse flies, respectively). *O. biloba* was highly host specific and almost exclusively recorded in barn swallows *Hirundo rustica* , except for single specimens collected from two passerine species (Savi's warbler and marsh warbler *Acrocephalus palustris* ). In contrast, *O. chloropus* was more generalist and recorded in nine host species, representing both passerines (three species, most prevalent in the common reed warbler *Acrocephalus scirpaceus* ) and nonpasserines (six species, most prevalent in the common snipe *Gallinago gallinago* ). Cervid‐associated *Lipoptena* species were recorded only occasionally (1.6% of all collected louse flies).

### 3.2 Associations of Total Louse Fly Prevalence With Host Traits and Environmental Factors

**FIGURE 3:**
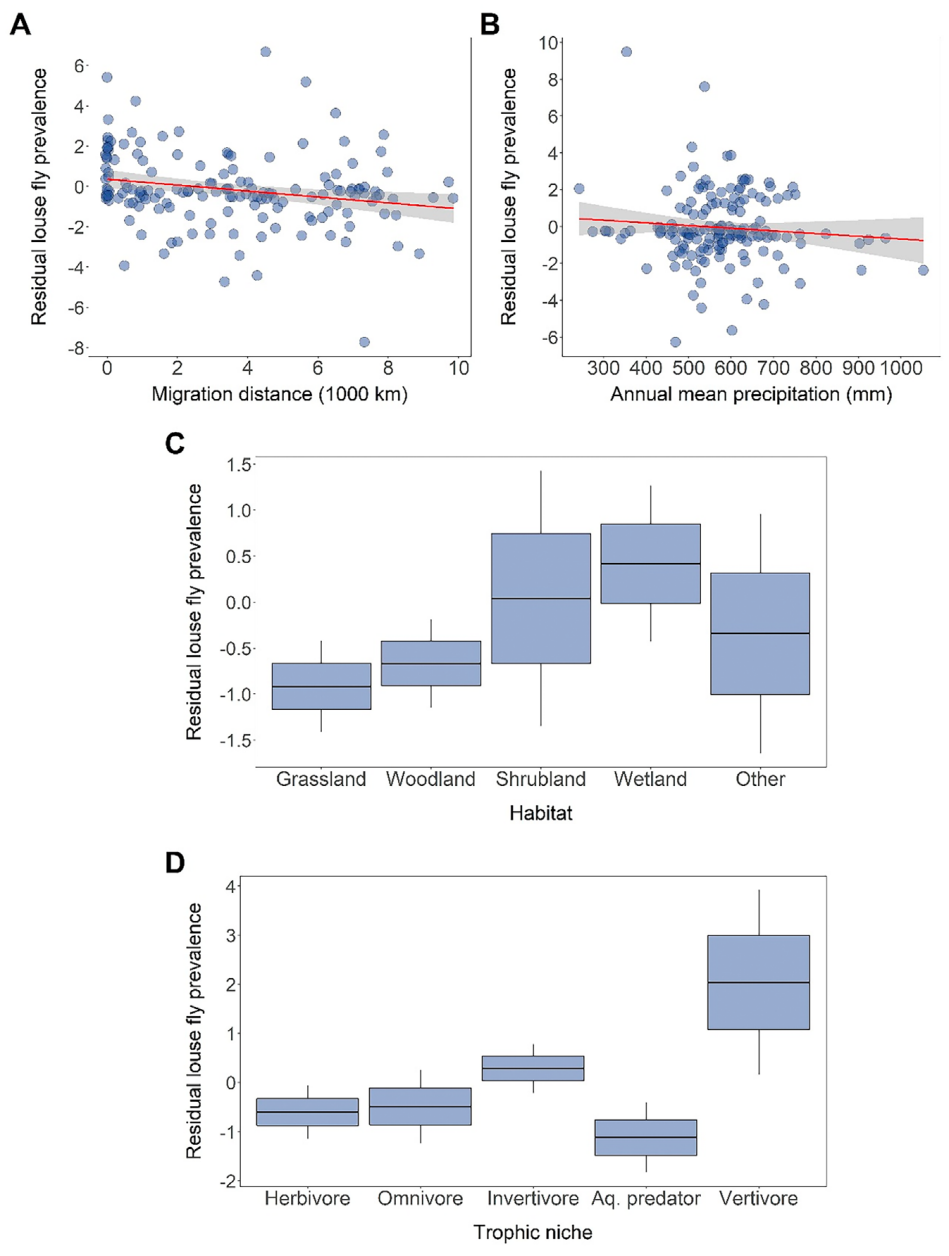
Associations of residual louse fly prevalence with host migration distance (A), annual mean precipitation (B), breeding habitat (C) and trophic niche (D), as assessed with phylogenetically informed Bayesian mixed models (model results shown in Table 1). Regression lines with 95% confidence intervals (grey ribbon) are shown in panels A and B, while means (central point), SE (box) and 95% confidence intervals (whiskers) are shown in panels C and D.

Phylogenetically informed analysis across the entire data set (*n* = 157 bird species) identified five host traits (body mass, migration distance, annual mean precipitation, habitat and trophic niche) as significant predictors of total louse fly prevalence (all louse fly species combined; Table 1). The remaining host‐related traits (age, date, clutch size, breeding latitude, annual mean temperature and nest placement) showed no significant association with louse fly prevalence and were removed from the full model (all *p* > 0.25; Table 1). In the reduced model, total louse fly prevalence was positively associated with host body mass, as larger bird species were more likely to be infected (*β* = 0.908; 95% CI: 0.212 to 1.678; *p* = 0.014; Table 1). Total louse fly prevalence was also significantly associated with migration distance (*β* = −0.0001; 95% CI: −0.0002 to 0.0000; *p* = 0.020; Table 1; Figure 3A), indicating that the louse fly prevalence decreased with the migration distance of their bird host species. Louse fly prevalence decreased significantly with an increase in the annual mean precipitation within the host breeding range (*β* = −0.004; 95% CI: −0.007 to −0.002; *p* = 0.002; Table 1; Figure 3B). Habitat type emerged as another significant predictor, since bird host species associated with woodland and grassland habitats showed lower prevalence compared to wetland birds (woodland: *β* = −1.181; 95% CI: −1.900 to −0.521; *p* = 0.002; grassland: *β* = −1.258; 95% CI: −2.481 to −0.166; *p* = 0.030; Table 1; Figure 3C). Finally, louse fly prevalence was significantly lower in omnivore than vertivore bird species (*β* = −1.507; 95% CI: −2.839 to −0.200; *p* = 0.040; Table 1; Figure 3D), indicating that the probability of infestation was related to host foraging strategy or diet.

**TABLE 1:** Associations of total louse fly prevalence with host traits among migrating birds (full data set: *N* = 157 avian species).

Predictor	Estimate	Lower 95% CL	Upper 95% CL	*p*
Full model				
Intercept	−2.660	−6.622	1.328	0.190
Age (proportion adult birds)	0.169	−1.436	1.888	0.832
Date	0.005	−0.006	0.015	0.356
Log body mass	0.669	0.015	1.565	0.072
Log clutch size	−0.299	−1.283	0.642	0.542
Breeding latitude	0.013	−0.018	0.044	0.422
**Migration distance**	**−0.0001**	**−0.0003**	**0.000**	**0.020**
Annual mean temperature	0.004	−0.005	0.012	0.356
**Annual mean precipitation**	**−0.005**	**−0.008**	**−0.0015**	**0.008**
Habitat: Grassland	−1.203	−2.576	−0.043	0.052
Habitat: Other	−0.360	−1.501	0.707	0.532
Habitat: Shrubland	−0.518	−1.337	0.390	0.236
**Habitat: Woodland**	**−1.021**	**−1.920**	**−0.353**	**0.016**
Trophic niche: Aquatic predator	−1.602	−3.560	0.212	0.118
Trophic niche: Herbivore	−1.360	−2.945	0.537	0.140
Trophic niche: Invertivore	−0.931	−2.289	0.29	0.176
**Trophic niche: Omnivore**	**−1.652**	**−3.017**	**−0.135**	**0.040**
Nest: Open elevated	0.282	−0.36	0.901	0.366
Nest: Open ground	0.351	−0.75	1.500	0.556
Reduced model				
Intercept	−1.937	−5.257	1.403	0.238
**Log body mass**	**0.908**	**0.212**	**1.678**	**0.014**
**Migration distance**	**−0.0001**	**−0.0002**	**0.000**	**0.020**
**Annual mean precipitation**	**−0.004**	**−0.007**	**−0.002**	**0.002**
**Habitat: Grassland**	**−1.258**	**−2.481**	**−0.166**	**0.030**
Habitat: Other	−0.733	−2.830	0.330	0.174
Habitat: Shrubland	−0.445	−1.226	0.600	0.326
**Habitat: Woodland**	**−1.181**	**−1.900**	**−0.521**	**0.002**
Trophic niche: Aquatic predator	−1.727	−3.395	0.371	0.106
Trophic niche: Herbivore	−1.217	−2.800	0.376	0.146
Trophic niche: Invertivore	−0.910	−2.066	0.294	0.150
**Trophic niche: Omnivore**	**−1.507**	**−2.839**	**−0.200**	**0.040**

*Note:* Coefficient estimates and corresponding 95% credibility limits (CL) were derived from phylogenetically informed Bayesian mixed models (full and reduced). Significant coefficients are bolded.

Most of the significant associations were retained in the model based on the subsampled data (species with small sample sizes excluded). Specifically, mean annual precipitation, habitat, and trophic niche were still identified as significant predictors of total louse fly prevalence, while the body mass and migration distance remained marginally significant (Table S1). At the same time, we found no evidence for significant directional changes in total louse fly prevalence over the sampling period or for significant differences in total louse fly prevalence among years (all *p* > 0.05, Table S2).

### 3.3 Host Traits and Environmental Factors Aspredictors of *O. avicularia* and *O. fringillina* Prevalence

**FIGURE 4:**
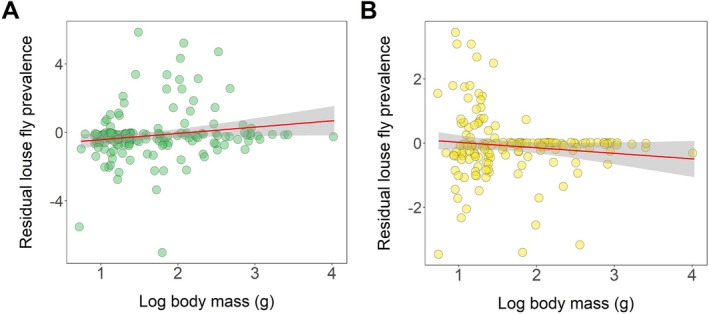
Associations of residual *Ornithomya avicularia* (A) and *O. fringillina* (B) prevalence with host body mass, as assessed with Bayesian mixed models (model results shown in Tables 2 and 3). Regression lines with 95% confidence intervals (grey ribbon) are shown.

Separate models for the two most common louse fly species revealed contrasting associations of host traits with raw prevalence of *O. avicularia* and *O. fringillina*. Host body mass was positively associated with the prevalence of *O. avicularia* (*β* = 2.499; 95% CI: 1.495 to 3.527; *p* < 0.001; Table 2; Figure 4A) but negatively associated with the prevalence of *O. fringillina* (*β* = −1.914; 95% CI: −3.189 to −0.630; *p* < 0.010; Table 3; Figure 4B). Annual mean precipitation was negatively associated with *O. avicularia* prevalence (*β* = −0.006; 95% CI: −0.010 to −0.001; *p* = 0.004) but not with *O. fringillina* prevalence (Tables 2 and 3). In contrast, migration distance was identified as a significant predictor of *O. fringillina* prevalence (lower prevalence with increasing migration distance; *β* = −0.0002; 95% CI: −0.0003 to −0.00005; *p* = 0.010; Table 3) but showed no significant association with *O. avicularia* prevalence (Table 2). Host habitat was identified as the only predictor showing a similar effect across the two louse fly species, as both *O. avicularia* and *O. fringillina* were less prevalent in hosts from woodland than wetland habitats (*O. avicularia*: *β* = −1.088; 95% CI: −2.136 to −0.002; *p* = 0.050; *O. fringillina*: *β* = −2.288; 95% CI: −3.200 to −1.376; *p* < 0.001; Tables 2 and 3). However, the prevalence of *O. fringillina* was also significantly lower in hosts from other (coastal, rock and human‐modified) habitats than wetlands (*β* = −2.597; 95% CI: −4.937 to −0.443; *p* = 0.010; Table 3). Other predictors (age, date, clutch size, breeding latitude, annual mean temperature, trophic niche and nest placement) did not show significant associations with the prevalence of either species (*p* > 0.05; Tables 2 and 3).

**TABLE 2:** Associations of *Ornithomya avicularia* prevalence with host traits among migrating birds (full data set: *N* = 157 avian species).

Predictor	Estimate	Lower 95% CL	Upper 95% CL	*p*
Full model				
Intercept	−4.322	−10.050	0.774	0.110
Age (proportion adult birds)	−0.393	−2.767	2.051	0.796
Date	−0.005	−0.022	0.014	0.600
**Log body mass**	**2.454**	**1.323**	**3.557**	**< 0.001**
Log clutch size	−0.577	−2.009	0.911	0.446
Breeding latitude	0.012	−0.041	0.074	0.700
Migration distance	−0.0002	−0.0004	−0.00001	0.052
Annual mean temperature	0.003	−0.011	0.018	0.748
**Annual mean precipitation**	**−0.006**	**−0.012**	**−0.0005**	**0.028**
Habitat: Grassland	−1.142	−3.327	0.501	0.202
Habitat: Other	−0.630	−2.786	1.471	0.552
Habitat: Shrubland	−0.555	−2.037	1.074	0.530
Habitat: Woodland	−0.958	−2.269	0.508	0.216
Trophic niche: Aquatic predator	−1.705	−4.492	0.716	0.186
Trophic niche: Herbivore	−1.148	−3.534	1.369	0.366
Trophic niche: Invertivore	−0.604	−2.511	1.023	0.492
Trophic niche: Omnivore	−1.141	−3.062	0.677	0.226
Nest: Open elevated	1.020	−0.227	2.343	0.108
Nest: Open ground	1.495	−0.361	3.371	0.126
Reduced model				
Intercept	−5.662	−10.590	−1.433	0.032
**Log body mass**	**2.499**	**1.495**	**3.527**	**< 0.001**
Migration distance	−0.0002	−0.0004	0.00003	0.070
**Annual mean precipitation**	**−0.006**	**−0.010**	**−0.001**	**0.004**
Habitat: Grassland	−1.281	−2.973	0.707	0.166
Habitat: Other	−0.593	−2.760	1.268	0.584
Habitat: Shrubland	−0.444	−1.910	0.963	0.534
**Habitat: Woodland**	**−1.088**	**−2.136**	**−0.002**	**0.050**
Trophic niche: Aquatic predator	−1.680	−4.276	0.753	0.180
Trophic niche: Herbivore	−1.160	−3.817	1.059	0.358
Trophic niche: Invertivore	−0.624	−2.170	1.082	0.432
Trophic niche: Omnivore	−1.111	−2.955	0.601	0.198
Nest: Open elevated	1.067	0.005	2.315	0.068
Nest: Open ground	1.407	−0.393	3.234	0.126

*Note:* Coefficient estimates and corresponding 95% credibility limits (CL) were derived from phylogenetically informed Bayesian mixed models (full and reduced). Significant coefficients are bolded.

**TABLE 3:** Associations of *Ornithomya fringillina* prevalence with host traits among migrating birds (full data set: *N* = 157 avian species).

Predictor	Estimate	Lower 95% CL	Upper 95% CL	*p*
Full model				
Intercept	−1.098	−6.030	4.007	0.680
Age (proportion adult birds)	−0.125	−2.798	2.498	0.952
Date	0.001	−0.015	0.017	0.892
**Log body mass**	**−2.406**	**−3.983**	**−0.979**	**0.010**
Log clutch size	−0.861	−2.296	0.723	0.254
Breeding latitude	0.026	−0.009	0.065	0.140
**Migration distance**	**−0.0002**	**−0.0004**	**−0.00003**	**0.016**
Annual mean temperature	0.008	−0.007	0.023	0.300
Annual mean precipitation	−0.005	−0.011	0.001	0.106
Habitat: Grassland	0.329	−1.689	2.548	0.794
Habitat: Other	−2.090	−5.129	0.689	0.094
Habitat: Shrubland	−0.214	−1.392	0.913	0.688
**Habitat: Woodland**	**−1.696**	**−3.004**	**−0.551**	**0.014**
Trophic niche: Aquatic predator	−3.278	−9.774	1.957	0.228
Trophic niche: Herbivore	1.780	−1.054	5.164	0.258
Trophic niche: Invertivore	1.705	−0.878	5.024	0.230
Trophic niche: Omnivore	1.617	−1.351	4.648	0.268
Nest: Open elevated	0.178	−0.824	1.145	0.734
Nest: Open ground	−0.174	−1.868	1.709	0.880
Reduced model				
Intercept	−2.130	−6.501	1.645	0.352
**Log body mass**	**−1.914**	**−3.189**	**−0.630**	**< 0.001**
Breeding latitude	0.013	−0.015	0.043	0.360
**Migration distance**	**−0.0002**	**−0.0003**	**−0.00005**	**0.010**
Annual mean precipitation	−0.002	−0.006	0.001	0.180
Habitat: Grassland	−0.401	−1.918	1.245	0.616
**Habitat: Other**	**−2.597**	**−4.937**	**−0.443**	**0.010**
Habitat: Shrubland	−0.451	−1.581	0.488	0.356
**Habitat: Woodland**	**−2.288**	**−3.200**	**−1.376**	**< 0.001**
Trophic niche: Aquatic predator	−3.794	−7.836	0.389	0.074
Trophic niche: Herbivore	1.304	−1.261	4.258	0.376
Trophic niche: Invertivore	1.109	−1.283	3.950	0.414
Trophic niche: Omnivore	1.105	−1.514	3.936	0.440

*Note:* Coefficient estimates and corresponding 95% credibility limits (CL) were derived from phylogenetically informed Bayesian mixed models (full and reduced). Significant coefficients are bolded.

Associations for body mass, annual mean precipitation and habitat retained significance in the analysis of identification‐adjusted prevalence (Tables S3 and S4), indicating little effect of unsampled/unidentified louse fly species on the obtained results. Analyses limited to species with at least ten host individuals screened (reduced model) yielded largely consistent results, supporting the significance and directionality of associations detected for both *O. avicularia* (body mass and annual precipitation; Table S5) and *O. fringillina* (body mass, migration distance, and habitat type; Table S6). At the same time, the models on subsampled data showed significant effects of nest placement on *O. avicularia* prevalence (higher prevalence in open elevated than cavity nests; *β* = 1.187; 95% CI: 0.000 to 2.350; *p* = 0.046; Table S5) and annual mean precipitation on *O. fringillina* prevalence (lower prevalence in hosts exposed to more precipitation; *β* = −0.006; 95% CI: −0.012 to 0.000; *p* = 0.044; Table S6).

## 4 Discussion

Our broad‐scale comparative analysis provided novel insights into how life‐history traits and ecological niches of host species shape ectoparasite (louse fly) occurrence across divergent bird taxa. Approximately 70% of bird species were infested with louse flies, with the highest prevalence observed in owls. Two louse fly species, *O. fringillina* (showing high specificity to small passerines) and *O. avicularia* (more generalist), were dominant across the dataset. Phylogenetically informed analyses revealed that the overall louse fly prevalence was significantly associated with several host‐related traits: It decreased with migration distance and annual precipitation within host breeding range, but increased with larger host body size and in certain habitat types. Moreover, omnivorous bird species showed significantly lower infestation probabilities than vertivores, reinforcing the role of host foraging strategy or diet in louse fly transmission. Separate models for the two dominant louse flies highlighted contrasting associations with host traits, suggesting divergent ecological strategies between *O. fringillina* and *O. avicularia*. These findings contribute to a more comprehensive picture of parasite dynamics in migratory bird populations and highlight the complex interplay between host biology and parasite ecology.

It has long been recognised that host species body size is a major determinant of parasite prevalence, since larger hosts offer a greater space (surface), higher resource availability and wider microhabitat (niche) diversity for ectoparasites (Poulin 1995; Clayton and Walther 1997). This may not only increase the richness of parasitic communities but also facilitate parasite reproduction and survival, hence increasing their abundance and transmission. Among avian hosts, species with larger body mass are often more heavily infested with ectoparasites, likely because their size increases detectability by parasites and reduces the efficiency of host grooming (Chu et al. 2019). Similar patterns have been reported in other host–parasite systems (Shuai et al. 2022; Henriksen et al. 2023), including the yellow‐necked mouse *Apodemus flavicollis* , where larger individuals carried higher loads of fleas and ticks (Zduniak et al. 2023). Our analyses revealed that host species‐level mean body mass was an important positive predictor of total louse fly prevalence. At the same time, we found that different louse fly species showed contrasting associations with host species body mass. A strong positive relationship was detected in *O. avicularia*, a generalist species that appears to effectively exploit larger hosts. The stronger association of *O. avicularia* with larger‐bodied hosts may reflect constraints related to parasite body size and life history. As a relatively large avian louse fly, *O. avicularia* may not be able to exploit small passerines efficiently, either because limited space and resources on the host reduce its survival or because it is more easily detected and removed by host grooming. In contrast, *O. fringillina*, exhibited a negative association with host body mass, being consistent with specialisation toward infecting small passerines. Consequently, this species appears less constrained by host morphology and size, which could be explained by its smaller structural dimensions or greater ecological flexibility. In fact, within our data set, the largest hosts (e.g., long‐eared owl, western marsh harrier *Circus aeruginosus*, or Eurasian sparrowhawk *Accipiter nisus* ) were often parasitised exclusively by *O. avicularia*, while the smallest hosts (e.g., goldcrest *Regulus regulus* and firecrest *Regulus ignicapilla* ) were only infected by O. *fringillina*. These differences highlight that even among generalist species, host–parasite associations can be shaped by species‐specific morphological and ecological traits.

Climatic conditions, particularly humidity and precipitation, are well known to influence the ecology of ectoparasitic insects, as moderate humidity often enhances parasite survival by reducing desiccation risk (Marshall 1981). While adult louse flies are expected to experience relatively constant microclimatic conditions in the plumage on their homeothermic avian hosts, overwintering puparia and free‐flying adults in search of the host may be vulnerable to adverse abiotic conditions, including temperature, humidity and precipitation encountered outside the hosts (Harkonen et al. 2012; Kaunisto et al. 2015). Our analyses indicated that annual precipitation was negatively associated with louse fly prevalence. Rain, often associated with lower temperatures, is known to markedly reduce the mobility of insects, particularly by hindering their ability to fly. For poor fliers, such as louse flies, it can significantly impair host‐finding, thereby increasing mortality. It is also likely that soaked feathers provide a much poorer refuge for louse flies, making them more easily detected by birds. Moreover, the drying and preening of wet feathers by birds may further increase the likelihood of louse flies being detected and removed by the host.

Habitat type was another important determinant of louse fly prevalence. Our results indicate that habitat type significantly influenced infestation rates, with woodland and grassland species showing lower prevalence compared to hosts associated with wetlands. This pattern was consistent across *O. avicularia* and *O. fringillina*, suggesting that the effect is not species‐specific, but instead reflects a general trend of reduced louse fly prevalence in nonwetland environments. A similar relationship was also observed for the total prevalence across all louse fly species. The lower occurrence of louse flies in woodlands may be linked to reduced host encounter rates or less favourable microclimatic conditions for parasite persistence compared to wetland habitats (Chahad‐Ehlers et al. 2018; van Hoesel et al. 2020). Interestingly, Lehikoinen et al. (2021) reported the opposite pattern, where *O. fringillina* preferred hosts associated with forest habitats, whereas all three (*O. avicularia, O. chloropus, O. fringillina*) louse fly species tended to avoid wetland‐associated hosts. This remarkable discrepancy suggests that associations of louse flies with hosts from varying habitats may be strongly dependent on the ecological or environmental context. Specifically, these contrasting patterns could either reflect variation across different stages of the host annual cycle, that is, reproductive season (the study by Lehikoinen et al. 2021 included data from breeding birds) vs. migration (our study), or geographic variation, with distinct patterns in Fennoscandia compared to Central Europe.

Trophic niche was also associated with louse fly prevalence, as host species classified as vertivores exhibited significantly higher infestation rates compared to omnivores. In general, predators feeding on a wide range of smaller prey tend to accumulate more parasites, due to increased opportunities for cross‐species transmission (Orlofske et al. 2015; McDevitt‐Galles et al. 2021). Hence, it is possible that avian raptors may acquire a variety of louse flies directly from their prey. Also, predators are generally larger than species occupying other trophic niches, and thus they can offer ectoparasites a broader range of habitats and resources, thus increasing their total prevalence. This highlights the importance of host trophic niche heterogeneity for parasite transmission, suggesting that both foraging mode and prey diversity may shape host–parasite associations in natural populations. By contrast, when examining individual louse fly species, trophic niche did not emerge as a significant predictor of prevalence. Both *O. avicularia* and *O. fringillina* showed stronger associations with host body size and habitat type rather than host diet, suggesting that structural and ecological traits of hosts are more relevant determinants of infestation risk by particular louse fly species than host foraging strategy.

Migration distance emerged as another significant predictor of louse fly prevalence. The prevalence decreased with increasing migration distance, consistent with the idea that long‐distance movements may disrupt parasite transmission cycles and reduce infestation through mechanical loss or mortality of ectoparasites during prolonged flights (Jenkins et al. 2012). This pattern was particularly clear for *O. fringillina*, which showed significantly lower prevalence with increasing migration distance. This species is closely associated with small passerines, including those migrating to sub‐Saharan Africa, such as the wetland species Savi's Warbler, Eurasian Reed Warbler and Great Reed Warbler. Their long migration distance may impose a strong constraint on ectoparasite persistence. However, in our study, *O. fringillina* were most common in short‐distance wetland migrants, such as the bearded reedling, and in owls, which migrate only within Europe. It can explain the negative relationship between this parasite prevalence and the host migration distance. By contrast, the prevalence of *O. avicularia* showed only a marginally significant relationship with the migration distance, which may reflect its ability to exploit both resident and short‐distance migrant hosts across a wide ecological range. It is also possible that short‐distance migrants not only retain parasites more effectively, but also contribute to their local transmission by scattering puparia produced continuously by females until autumn (Janiszewska et al. 2025). Resident birds tend to use the same areas and habitats for breeding and foraging outside the breeding season, which may increase their exposure to louse fly puparia and boost local transmission cycles. Lehikoinen et al. (2021) did not detect any associations between louse flies and migration distance, suggesting that such relationships may become more evident during the migration period itself (as observed in our study).

Host nest type did not predict overall louse fly prevalence in our data set, but we detected species‐specific responses of louse flies to host nest placement. Specifically, *O. avicularia* tended to be more prevalent in hosts that use open elevated nests for breeding, suggesting that exposed nesting environments may facilitate parasite transmission by increasing accessibility to different hosts and contact rates among individuals. This result is consistent with earlier studies showing that nest architecture influences ectoparasite colonisation and host–parasite interactions (Heeb et al. 2000). In contrast, *O. fringillina* did not exhibit any association with nest type, indicating that its prevalence is more strongly determined by host size and habitat than by its breeding ecology. Consistent with our results, Lehikoinen et al. (2021) reported a preference of *O. avicularia* for hosts breeding in open tree nests. However, these analyses did not rely on the prevalence data but were limited to the comparison of the proportions of host (and nonhost) species using different nest types. Our comparative analysis adds to the previous research, showing that open nesting birds are not only preferred as hosts by *O. avicularia*, but open nesting also increases *O. avicularia* prevalence, likely due to enhanced transmission.

Despite robust data (over 100,000 individuals screened), some limitations of our study should be acknowledged. By focusing only on the autumn migration period, our study may not capture peak parasite prevalence occurring in other seasons. For instance, research on avian haemosporidian parasites has consistently shown higher infestation intensities in summer and spring, both during breeding and stopover periods (Šujanová et al. 2021; Han et al. 2023). Similarly, seasonal profiles of generalist ecto‐ and endoparasites indicate marked fluctuations across the annual cycle, with microhabitat changes during the reproductive season playing a key role (e.g., *I. ricinus* and *I. arboricola* infestation rates dropping from ca 21.6% during breeding to 6.8% in the nonbreeding period) (Kocianová et al. 2017). As a result, our estimates of louse fly prevalence may not be easily extrapolatable to prebreeding or breeding seasons, when host nesting behaviour enhances transmission opportunities. On the other hand, louse flies that attach to a host during the nesting period usually remain with it until the end of their lives, and at least infestation by most females (which live longer than males; Kemper 1951) should well extend into the autumn period, when we conducted sampling. Nevertheless, incorporating sampling over different seasons would provide a more comprehensive picture of host–parasite dynamics.

In conclusion, our findings provide novel evidence on the ecological and environmental factors shaping louse fly distribution in birds using European migratory flyways. Our results indicate that louse fly prevalence during autumn migration is influenced not only by morphological and ecological host traits, such as body size, habitat or trophic niche, but also by annual climatic conditions within the host breeding range. These results contribute to the growing understanding of host–parasite interactions in highly dynamic biological systems shaped by the extreme mobility of host organisms. We recommend that future studies aim to collect and incorporate data from across the entire host annual cycle and across multiple geographic regions, as well as examine potential fitness costs of infestation by louse flies. Combining ecological, behavioural and physiological data is likely to further advance our understanding of the role of ectoparasites, such as louse flies, in shaping migratory strategies and host life histories.

Migration strategy and climatic conditions can serve as valuable predictors of parasite susceptibility across species. Ongoing climate change is expected to alter both the distribution of ectoparasites and the migratory patterns of their avian hosts, potentially reshaping host–parasite interactions across time and space (Møller et al. 2013; Mennerat et al. 2021). Modifications in migration tactics, such as shorter routes, altered timing or prolonged stopovers, may increase exposure to novel parasite assemblages or extend the duration of host–parasite contact (Hegemann et al. 2022). Because parasite pressure can exacerbate existing environmental and demographic threats, it is crucial to identify the traits that make individual birds or species more susceptible to ectoparasite infections. Such knowledge is particularly important for conservation efforts, as understanding parasite‐mediated risks can help predict and mitigate the accumulating impacts of climate change on vulnerable bird populations.

## Author Contributions

**Aleksandra Janiszewska:** conceptualization (lead), data curation (lead), formal analysis (lead), investigation (equal), methodology (equal), resources (equal), writing – original draft (lead). **Piotr Minias:** conceptualization (equal), data curation (lead), investigation (equal), resources (equal), writing – review and editing (lead). **Radosław Włodarczyk:** investigation (equal), resources (equal), writing – review and editing (equal). **Maciej Kamiński:** investigation (equal), resources (equal), writing – review and editing (equal). **Dariusz Jakubas:** investigation (equal), resources (equal), writing – review and editing (equal). **Magdalena Remisiewicz:** investigation (equal), resources (equal), writing – review and editing (equal). **Hanna Sztwiertnia:** investigation (equal), resources (equal), writing – review and editing (equal). **Maciej Bartos:** conceptualization (equal), data curation (equal), investigation (equal), resources (equal), supervision (lead), writing – original draft (equal), writing – review and editing (lead).

## Funding

The maintenance of Mierzeja Wiślana, Bukowo, and Druzno Lake stations was supported by the Special Research Facility grants (SPUB) from the Polish Ministry of Science and Higher Education to the University of Gdańsk.

## Ethics Statement

The fieldwork in the Jeziorsko and Druzno Lake nature reserves was conducted by the permissions from the Regional Directorate for Environmental Protection in Łódź and Olsztyn, respectively. The fieldwork at Mierzeja Wiślana and Bukowo stations was conducted by the permission from the General Directorate for Environmental Protection in Poland and was approved annually by the Marine Office in Gdynia and the Forestry Division in Elbląg (Mierzeja Wiślana) or Marine Office in Słupsk or Szczecin (Bukowo). All bird catching and ringing was conducted with an annual approval of the Polish Bird Ringing Centre of the Polish Academy of Sciences.

## Conflicts of Interest

The authors declare no conflicts of interest.

## Supporting information


**Table S1:** Associations of total louse fly prevalence with host traits among migrating birds (subsampled data set: *n* = 105 avian species with at least 10 individuals screened). Coefficient estimates and corresponding 95% credibility limits (CL) were derived from phylogenetically informed Bayesian mixed models (full and reduced). All categorical variables were coded relative to the following reference levels: Wetland for habitat, Vertivore for trophic niche and Cavity for nest type. Significant coefficients are marked in bold.
**Table S2:** Interannual variation in total louse fly prevalence of migrating birds (full data set: *n* = 157 avian species). Year was included either as a linear covariate to test for directional changes in prevalence over the sampling period (Model S2.1) or as a fixed factor to test for differences in prevalence among years (Model S2.2). Year 2014 was used as reference level. Coefficient estimates and corresponding 95% credibility limits (CL) were derived from phylogenetically informed Bayesian mixed models.
**Table S3:** Associations of identification‐adjusted prevalence of *Ornithomya avicularia* with host traits among migrating birds (full data set: *n* = 157 avian species). Coefficient estimates and corresponding 95% credibility limits (CL) were derived from phylogenetically informed Bayesian mixed models (full and reduced). Significant coefficients are bolded.
**Table S4:** Associations of identification‐adjusted prevalence of *Ornithomya fringillina* with host traits among migrating birds (full data set: *n* = 157 avian species). Coefficient estimates and corresponding 95% credibility limits (CL) were derived from phylogenetically‐informed Bayesian mixed models (full and reduced). Significant coefficients are bolded.
**Table S5:** Associations of *Ornithomya avicularia* prevalence with host traits among migrating birds (subsampled dataset: *n* = 105 avian species with at least 10 individuals screened). Coefficient estimates and corresponding 95% credibility limits (CL) were derived from phylogenetically informed Bayesian mixed models (full and reduced). All categorical variables were coded relative to the following reference levels: Wetland for habitat, Vertivore for trophic niche and Cavity for nest type. Significant coefficients are marked in bold.
**Table S6:** Associations of *Ornithomya fringillina* prevalence with host traits among migrating birds (subsampled dataset: *n* = 105 avian species with at least 10 individuals screened). Coefficient estimates and corresponding 95% credibility limits (CL) were derived from phylogenetically‐informed Bayesian mixed models (full and reduced). All categorical variables were coded relative to the following reference levels: Wetland for habitat, Vertivore for trophic niche and Cavity for nest type. Significant coefficients are marked in bold.

## Data Availability

The dataset used in this study is provided and is available as Supporting Information.

## References

[ece373846-bib-0001] Abad‐Franch, F. , F. A. Monteiro , M. G. Pavan , et al. 2021. “Under Pressure: Phenotypic Divergence and Convergence Associated With Microhabitat Adaptations in Triatominae.” Parasites & Vectors 14: 195. 10.1186/s13071-021-04647-z. 33832518 PMC8034103

[ece373846-bib-0002] Ajith, Y. , U. Dimri , E. Madhesh , D. Kumar , A. Gopalakrishnan , and M. C. Sharma . 2020. “Influence of Weather Patterns and Air Quality on Ecological Population Dynamics of Ectoparasites in Goats.” International Journal of Biometeorology 64, no. 10: 1731–1742. 10.1007/s00484-020-01952-7. 32556594

[ece373846-bib-0003] Babyesiza, W. S. , J. Mpagi , J. Ssuuna , S. Akoth , and A. Katakweba . 2023. “Ectoparasite Fauna of Rodents and Shrews With Their Spatial, Temporal and Dispersal Along a Degradation Gradient in Mabira Central Forest Reserve.” Journal of Parasitology Research 2023: 7074041. 10.1155/2023/7074041. 37928436 PMC10625493

[ece373846-bib-0004] BirdLife International & Handbook of the Birds of the World . 2016. “Bird Species Distribution Maps of The World (Version 6.0).” http://datazone.birdlife.org/species/requestdis.

[ece373846-bib-0005] Bivand, R. , and C. Rundel . 2023. “rgeos: Interface to Geometry Engine, Open Source (GEOS).” https://r‐forge.r‐project.org/projects/rgeos/.

[ece373846-bib-0006] Boeger, W. A. , D. C. Kritsky , and M. R. Pie . 2003. “Context of Diversification of the Viviparous Gyrodactylidae (Platyhelminthes, Monogenoidea).” Zoologica Scripta 32, no. 2: 159–172. 10.1046/j.1463-6409.2003.00130.x. PMC7165889 32336863

[ece373846-bib-0991] Borowiec, L. 1984. Klucze do oznaczania owadów Polski cz. XXVIII Muchówki – Diptera z. 77 Wpleszczowate – Hippoboscidae. PWN, PTEnt.

[ece373846-bib-0007] Brockhurst, M. A. , T. A. Chapman , K. C. King , J. E. Mank , S. Paterson , and G. D. D. Hurst . 2014. “Running With the Red Queen: The Role of Biotic Conflicts in Evolution.” Proceedings of the Royal Society B: Biological Sciences 281, no. 1797: 20141382. 10.1098/rspb.2014.1382. PMC4240979 25355473

[ece373846-bib-0008] Bush, S. E. , D. Kim , M. Reed , and D. H. Clayton . 2010. “Evolution of Cryptic Coloration in Ectoparasites.” American Naturalist 176, no. 4: 529–535. 10.1086/656269. 20722554

[ece373846-bib-0009] Chahad‐Ehlers, S. , A. T. Fushita , G. A. Lacorte , P. C. P. Assis , and S. N. del Lama . 2018. “Effects of Habitat Suitability for Vectors, Environmental Factors and Host Characteristics on the Spatial Distribution of the Diversity and Prevalence of Haemosporidians in Waterbirds From Three Brazilian Wetlands.” Parasites & Vectors 11: 276. 10.1186/s13071-018-2847-z. 29716645 PMC5930942

[ece373846-bib-0010] Chu, X. , C. Xia , C. Yang , W. Jiang , W. Liang , and B. G. Stokke . 2019. “The Influence of Host Body Size and Food Guild on Chewing Louse Prevalence in Birds.” Journal of Parasitology 105, no. 2: 301–305. 10.1645/17-137. 31021736

[ece373846-bib-0011] Clayton, D. H. , and J. Moore , eds. 2013. Host–Parasite Evolution: General Principles and Avian Models. Oxford University Press.

[ece373846-bib-0012] Clayton, D. H. , and B. A. Walther . 1997. “Collection and Quantification of Arthropod Parasites of Birds.” In Host–Parasite Evolution: General Principles and Avian Models, edited by D. H. Clayton and J. Moore , 419–440. Oxford University Press.

[ece373846-bib-0013] Dawkins, R. , and J. R. Krebs . 1979. “Arms Races Between and Within Species.” Proceedings of the Royal Society B: Biological Sciences 205, no. 1161: 489–511. 10.1098/rspb.1979.0081. 42057

[ece373846-bib-0014] del Hoyo, J. , A. Elliott , and J. Sargatal , eds. 1992–2011. Handbook of the Birds of the World. Vol. 1–16. Lynx Edicions.

[ece373846-bib-0015] Demongin, L. 2016. Identification Guide to Birds in the Hand. Pelagic Publications.

[ece373846-bib-0016] Dube, W. C. , A. K. Hund , S. P. Turbek , and R. J. Safran . 2018. “Microclimate and Host Body Condition Influence Mite Population Growth in a Wild Bird–Ectoparasite System.” International Journal for Parasitology: Parasites and Wildlife 7, no. 3: 301–308. 10.1016/j.ijppaw.2018.07.007. 30128287 PMC6097460

[ece373846-bib-0017] Dunning, J. B., Jr. 2008. CRC Handbook of Avian Body Masses. CRC Press.

[ece373846-bib-0018] Ericson, P. G. , C. L. Anderson , T. Britton , et al. 2006. “Diversification of Neoaves: Integration of Molecular Sequence Data and Fossils.” Biology Letters 2: 543–547. 10.1098/rsbl.2006.0523. 17148284 PMC1834003

[ece373846-bib-0019] Fain, A. 1994. “Adaptation, Specificity and Host–Parasite Coevolution in Mites (Acari).” International Journal for Parasitology 24, no. 8: 1273–1283. 10.1016/0020-7519(94)90194-5. 7729980

[ece373846-bib-0020] Fick, S. E. , and R. J. Hijmans . 2017. “WorldClim 2: New 1‐Km Spatial Resolution Climate Surfaces for Global Land Areas.” International Journal of Climatology 37: 4302–4315. 10.1002/joc.5086.

[ece373846-bib-0021] Franke, F. , S. A. O. Armitage , M. A. M. Kutzer , J. Kurtz , and J. P. Scharsack . 2017. “Environmental Temperature Variation Influences Fitness Trade‐Offs and Tolerance in a Fish–Tapeworm Association.” Parasites & Vectors 10: 252. 10.1186/s13071-017-2192-7. 28571568 PMC5455083

[ece373846-bib-0022] Gebrezgiher, G. B. , R. H. Makundi , A. A. S. Katakweba , S. R. Belmain , C. M. Lyimo , and Y. Meheretu . 2023. “Arthropod Ectoparasites of Two Rodent Species Occurring in Varied Elevations on Tanzania's Second Highest Mountain.” Biology 12, no. 3: 394. 10.3390/biology12030394. 36979086 PMC10045264

[ece373846-bib-0023] Geweke, J. 1992. “Evaluating the Accuracy of Sampling‐Based Approaches to the Calculation of Posterior Moments.” In Bayesian Statistics, edited by J. M. Bernardo , A. P. Berger , A. P. Dawid , and A. F. M. Smith , vol. 4, 169–193. Oxford University Press.

[ece373846-bib-0024] Hadfield, J. D. 2010. “MCMC Methods for Multi‐Response Generalized Linear Mixed Models: The *MCMCglmm* R Package.” Journal of Statistical Software 33: 1–22. 20808728 PMC2929880

[ece373846-bib-0025] Hadfield, J. D. , and S. Nakagawa . 2010. “General Quantitative Genetic Methods for Comparative Biology: Phylogenies, Taxonomies and Multi‐Trait Models for Continuous and Categorical Characters.” Journal of Evolutionary Biology 23: 494–508. 10.1111/j.1420-9101.2009.01915.x. 20070460

[ece373846-bib-0026] Hall, A. R. , P. D. Scanlan , A. D. Morgan , and A. Buckling . 2011. “Host–Parasite Coevolutionary Arms Races Give Way to Fluctuating Selection.” Ecology Letters 14, no. 7: 635–642. 10.1111/j.1461-0248.2011.01624.x. 21521436

[ece373846-bib-0027] Han, Y. , O. Hellgren , Q. Wu , et al. 2023. “Seasonal Variations of Intensity of Avian Malaria Infection in the Thousand Island Lake System, China.” Parasites & Vectors 16, no. 1: 218. 10.1186/s13071-023-05848-4. 37403099 PMC10318837

[ece373846-bib-0028] Harkonen, L. , A. Kaitala , S. Kaunisto , and T. Repo . 2012. “High Cold Tolerance Through Four Seasons and All Free‐Living Stages in an Ectoparasite.” Parasitology 139, no. 7: 926–933. 10.1017/S0031182012000091. 22313619

[ece373846-bib-0029] Heath, A. C. 2020. “Climate Change and Its Potential for Altering the Phenology and Ecology of Some Common and Widespread Arthropod Parasites in New Zealand.” New Zealand Veterinary Journal 69, no. 1: 5–19. 10.1080/00480169.2020.1787276. 32586220

[ece373846-bib-0030] Heeb, P. , M. Kölliker , and H. Richner . 2000. “Bird‐Ectoparasite Interactions, Nest Humidity, and Ectoparasite Community Structure.” Ecology 81, no. 4: 958–968. 10.2307/177170.

[ece373846-bib-0031] Hegemann, A. , C. Birberg , D. Hasselquist , and J.‐Å. Nilsson . 2022. “Early and Late Migrating Avian Individuals Differ in Constitutive Immune Function and Blood Parasite Infections, but Patterns Depend on the Migratory Strategy.” Frontiers in Ecology and Evolution 10: 880426. 10.3389/fevo.2022.880426.

[ece373846-bib-0032] Henriksen, E. H. , A. Frainer , R. Poulin , R. Knudsen , and P. A. Amundsen . 2023. “Ectoparasite Population Dynamics Are Affected by Host Body Size but Not Host Density or Water Temperature in a 32‐Year Long Time Series.” Oikos 2023: e09328. 10.1111/oik.09328.

[ece373846-bib-0033] Holand, H. , H. Jensen , T. Kvalnes , et al. 2019. “Parasite Prevalence Increases With Temperature in an Avian Metapopulation in Northern Norway.” Parasitology 146, no. 8: 1030–1035. 10.1017/S0031182019000337. 30977457

[ece373846-bib-0034] Hutson, A. M. 1984. Diptera: Keds, Flat‐Flies & Bat‐Flies (Hippoboscidae & Nycteribiidae). Royal Entomological Society of London.

[ece373846-bib-0035] Janiszewska, A. , T. Rewicz , P. Minias , et al. 2025. “Host‐Related Genetic Differentiation of a Polyxenic Avian Ectoparasite, *Ornithomya avicularia* (Hippoboscidae).” International Journal for Parasitology: Parasites and Wildlife 27: 101081. 10.1016/j.ijppaw.2025.101081. 40487347 PMC12141070

[ece373846-bib-0036] Jenkins, T. , G. H. Thomas , O. Hellgren , and I. P. F. Owens . 2012. “Migratory Behavior of Birds Affects Their Coevolutionary Relationship With Blood Parasites.” Evolution 66, no. 3: 740–751. 10.1111/j.1558-5646.2011.01470.x. 22380437

[ece373846-bib-0037] Jetz, W. , G. H. Thomas , J. B. Joy , K. Hartmann , and A. O. Mooers . 2021. “The BirdTree.org: A Global Phylogeny of Birds (Version 3.0).” http://birdtree.org.

[ece373846-bib-0038] Johnson, K. P. , S. M. Shreve , and V. S. Smith . 2012. “Repeated Adaptive Divergence of Microhabitat Specialization in Avian Feather Lice.” BMC Biology 10: 52. 10.1186/1741-7007-10-52. 22717002 PMC3391173

[ece373846-bib-0039] Kaunisto, S. , L. Harkonen , M. J. Rantala , and R. Kortet . 2015. “Early‐Life Temperature Modifies Adult Encapsulation Response in an Invasive Ectoparasite.” Parasitology 142, no. 10: 1290–1296. 10.1017/S0031182015000591. 26040308

[ece373846-bib-0040] Kemper, H. 1951. “Beobachtungen an *Crataerina pallida* Latr. und *Melophagus ovinus* L. (Diptera, Pupipara).” Zeitschrift für Hygiene (Zoologie) 39: 225–259.

[ece373846-bib-0041] Keve, G. , T. Csörgő , D. Kováts , et al. 2024. “Contributions to Our Knowledge of Avian Louse Flies (Hippoboscidae: Ornithomyinae) With the First European Record of the African Species *Ornithoctona laticornis* .” Parasites & Vectors 17: 237. 10.1186/s13071-024-06303-8. 38797857 PMC11129389

[ece373846-bib-0042] Kocianová, E. , V. Rusňáková Tarageľová , D. Haruštiaková , and E. Špitalská . 2017. “Seasonal Infestation of Birds With Immature Stages of *Ixodes ricinus* and *Ixodes arboricola* .” Ticks and Tick‐Borne Diseases 8, no. 3: 423–431. 10.1016/j.ttbdis.2017.01.006. 28169171

[ece373846-bib-0043] Kołodziej‐Sobocińska, M. 2019. “Factors Affecting the Spread of Parasites in Populations of Wild European Terrestrial Mammals.” Mammal Research 64: 301–318. 10.1007/s13364-019-00423-8.

[ece373846-bib-0044] Lehane, M. J. 2005. The Biology of Blood‐Sucking in Insects. Cambridge University Press.

[ece373846-bib-0045] Lehikoinen, A. , P. Pohjola , J. Valkama , M. Mutanen , and J. L. O. Pohjoismäki . 2021. “Promiscuous Specialists: Host Specificity Patterns Among Generalist Louse Flies.” PLoS One 16, no. 5: e0247698. 10.1371/journal.pone.0247698. 34043636 PMC8158981

[ece373846-bib-0046] Marshall, A. G. 1981. The Ecology of Ectoparasitic Insects. Academic Press.

[ece373846-bib-0047] Mbora, D. N. M. , and M. A. McPeek . 2009. “Host Density and Human Activities Mediate Increased Parasite Prevalence and Richness in Primates Threatened by Habitat Loss and Fragmentation.” Journal of Animal Ecology 78: 210–218. 10.1111/j.1365-2656.2008.01481.x. 19120603

[ece373846-bib-0048] McDevitt‐Galles, T. , S. A. Carpenter , J. Koprivnikar , and P. T. J. Johnson . 2021. “How Predator and Parasite Size Interact to Determine Consumption of Infectious Stages.” Oecologia 197, no. 3: 551–564. 10.1007/s00442-021-05010-w. 34405300

[ece373846-bib-0049] Mennerat, A. , A. Charmantier , P. Perret , S. Hurtrez‐Boussès , and M. M. Lambrechts . 2021. “Parasite Intensity Is Driven by Temperature in a Wild Bird.” Peer Community Journal 1: e60. 10.24072/pcjournal.60.

[ece373846-bib-0050] Minias, P. , and R. Włodarczyk . 2020. “Avian Developmental Rates Are Constrained by Latitude and Migratoriness: A Global Analysis.” Journal of Biogeography 47, no. 10: 2218–2229. 10.1111/jbi.13923.

[ece373846-bib-0052] Møller, A. P. , S. Merino , J. J. Soler , et al. 2013. “Assessing the Effects of Climate on Host–Parasite Interactions: A Comparative Study of European Birds and Their Parasites.” PLoS One 8, no. 12: e82886. 10.1371/journal.pone.0082886. 24391725 PMC3876993

[ece373846-bib-0053] Moyer, B. R. , D. M. Drown , and D. H. Clayton . 2002. “Low Humidity Reduces Ectoparasite Pressure: Implications for Host Life History Evolution.” Oikos 97, no. 2: 223–228. 10.1034/j.1600-0706.2002.970208.x.

[ece373846-bib-0054] Mundry, R. , and C. L. Nunn . 2008. “Stepwise Model Fitting and Statistical Inference: Turning Noise Into Signal Pollution.” American Naturalist 173: 119–123. 10.1086/593303 19049440

[ece373846-bib-0055] Oboňa, J. , O. Sychra , S. Greš , et al. 2019. “A Revised Annotated Checklist of Louse Flies (Diptera, Hippoboscidae) From Slovakia.” ZooKeys 862: 129–152. 10.3897/zookeys.862.25992. 31341389 PMC6635380

[ece373846-bib-0056] Orlofske, S. A. , R. C. Jadin , and P. T. Johnson . 2015. “It's a Predator‐Eat‐Parasite World: How Characteristics of Predator, Parasite and Environment Affect Consumption.” Oecologia 178, no. 2: 537–547. 10.1007/s00442-015-3243-4. 25648648

[ece373846-bib-0057] Poulin, R. 1995. Evolutionary Ecology of Parasites: A Review of Parasitism in Wild Animal Populations. Chapman & Hall.

[ece373846-bib-0058] Poulin, R. 2007. Evolutionary Ecology of Parasites. 2nd ed. Princeton University Press.

[ece373846-bib-0059] Price, R. D. , R. A. Hellenthal , R. L. Palma , K. P. Johnson , and D. H. Clayton . 2003. The Chewing Lice: World Checklist and Biological Overview. Illinois Natural History Survey Special Publication.

[ece373846-bib-0060] Rewicz, T. , A. Móra , G. Tończyk , et al. 2021. “First Records Raise Questions: DNA Barcoding of Odonata in the Middle of the Mediterranean.” Genome 64, no. 3: 196–206. 10.1139/gen-2019-0226. 32502367

[ece373846-bib-0061] Roy, V. 2020. “Convergence Diagnostics for Markov Chain Monte Carlo.” Annual Review of Statistics and Its Application 7: 387–412. 10.1146/annurev-statistics-031219-041237.

[ece373846-bib-0062] Santolíková, A. , J. Brzoňová , I. Čepička , and M. Svobodová . 2022. “Avian Louse Flies and Their Trypanosomes: New Vectors, New Lineages and Host–Parasite Associations.” Microorganisms 10: 584. 10.3390/microorganisms10030584. 35336159 PMC8948672

[ece373846-bib-0063] Shilereyo, M. , F. Magige , P. S. Ranke , J. O. Ogutu , and E. Røskaft . 2022. “Ectoparasite Load of Small Mammals in the Serengeti Ecosystem: Effects of Land Use, Season, Host Species, Age, Sex and Breeding Status.” Parasitology Research 121, no. 3: 823–838. 10.1007/s00436-022-07439-1. 35122139 PMC8858283

[ece373846-bib-0064] Shuai, L. Y. , D. Wu , W. Wei , L. Xu , W. Wei , and D. H. Wang . 2022. “Ecological Correlates of Ectoparasite Load in a Rodent: Complex Roles of Seasonality.” Current Research in Parasitology & Vector‐Borne Diseases 2: 100057. 10.1016/j.crpvbd.2022.100057. PMC9253529 35800108

[ece373846-bib-0065] Snow, D. W. , and C. M. Perrins . 1998. The Birds of the Western Palearctic. Vol. 1–2. Concise ed. Oxford University Press.

[ece373846-bib-0066] Šujanová, A. , E. Špitalská , and R. Václav . 2021. “Seasonal Dynamics and Diversity of Haemosporidians in a Natural Woodland Bird Community in Slovakia.” Diversity 13, no. 9: 439. 10.3390/d13090439.

[ece373846-bib-0067] Tobias, J. A. , and A. L. Pigot . 2019. “Integrating Behaviour and Ecology Into Global Biodiversity Conservation Strategies.” Philosophical Transactions of the Royal Society B 374, no. 1781: 20190012. 10.1098/rstb.2019.0012. PMC6710563 31352893

[ece373846-bib-0068] Tobias, J. A. , C. Sheard , A. L. Pigot , et al. 2022. “AVONET: Morphological, Ecological and Geographical Data for All Birds.” Ecology Letters 25: 581–597. 10.1111/ele.13898. 35199922

[ece373846-bib-0069] Turcotte, A. , M. Bélisle , F. Pelletier , and D. Garant . 2018. “Environmental Determinants of Haemosporidian Parasite Prevalence in a Declining Population of Tree Swallows.” Parasitology 145, no. 7: 961–970. 10.1017/S0031182017002128. 29166965

[ece373846-bib-0070] van Hoesel, W. , D. Santiago‐Alarcon , A. Marzal , and S. C. Renner . 2020. “Effects of Forest Structure on the Interaction Between Avian Hosts, Dipteran Vectors and Haemosporidian Parasites.” BMC Ecology 20: 47. 10.1186/s12898-020-00315-5. 32814584 PMC7437053

[ece373846-bib-0071] Vázquez, D. P. , R. Poulin , B. R. Krasnov , and G. I. Shenbrot . 2005. “Species Abundance and the Distribution of Specialization in Host–Parasite Interaction Networks.” Journal of Animal Ecology 74, no. 5: 946–955. 10.1111/j.1365-2656.2005.00992.x.

[ece373846-bib-0072] Vilela, B. , and F. Villalobos . 2015. “letsR: A New R Package for Data Handling and Analysis in Macroecology.” Methods in Ecology and Evolution 6: 1229–1234. 10.1111/2041-210X.12401.

[ece373846-bib-0073] Zduniak, M. , S. Serafini , A. Wróbel , and R. Zwolak . 2023. “Host Body Mass, Not Sex, Affects Ectoparasite Loads in Yellow‐Necked Mouse *Apodemus flavicollis* .” Parasitology Research 122, no. 11: 2599–2607. 10.1007/s00436-023-07958-5. 37702846 PMC10567855

