# Supplementary material for: Louse Flies on the Fly: Host Macroecology Shapes Interspecific Variation in Ectoparasite Prevalence Among Migrating Birds
Source: Ecol Evol. 2026 Jun 18;16(6):e73846. doi: 10.1002/ece3.73846 (PMC13277761; doi:10.1002/ece3.73846)
Supplement: Supplementary file 1 — Table S1: Associations of total louse fly prevalence with host traits among migrating birds (subsampled data set: n = 105 avian species with at least 10 individuals screened). Coefficient estimates and corresponding 95% credibility limits (CL) were derived from phylogenetically informed Bayesian mixed models (full and reduced). All categorical variables were coded relative to the following reference levels: Wetland for habitat, Vertivore for trophic niche and Cavity for nest type. Significant coefficients are marked in bold. Table S2: Interannual variation in total louse fly prevalence of migrating birds (full data set: n = 157 avian species). Year was included either as a linear covariate to test for directional changes in prevalence over the sampling period (Model S2.1) or as a fixed factor to test for differences in prevalence among years (Model S2.2). Year 2014 was used as reference level. Coefficient estimates and corresponding 95% credibility limits (CL) were derived from phylogenetically informed Bayesian mixed models. Table S3: Associations of identification‐adjusted prevalence of Ornithomya avicularia with host traits among migrating birds (full data set: n = 157 avian species). Coefficient estimates and corresponding 95% credibility limits (CL) were derived from phylogenetically informed Bayesian mixed models (full and reduced). Significant coefficients are bolded. Table S4: Associations of identification‐adjusted prevalence of Ornithomya fringillina with host traits among migrating birds (full data set: n = 157 avian species). Coefficient estimates and corresponding 95% credibility limits (CL) were derived from phylogenetically‐informed Bayesian mixed models (full and reduced). Significant coefficients are bolded. Table S5: Associations of Ornithomya avicularia prevalence with host traits among migrating birds (subsampled dataset: n = 105 avian species with at least 10 individuals screened). Coefficient estimates and corresponding 95% credibility limits ( [file ECE3-16-e73846-s001.docx]

**Electronic Supplementary Material**

**Louse flies on the fly: host macroecology shapes inter-specific variation in ectoparasite prevalence among migrating birds**

**Aleksandra Janiszewska*^a^, Piotr Minias^a^, Radosław Włodarczyk^a^, Maciej Kamiński^a^, Dariusz Jakubas^b^, Magdalena Remisiewicz^c^, Hanna Sztwiertnia^d^, Maciej Bartos*^a^**

*^a^University of Lodz, Faculty of Biology and Environmental Protection, Department of Biodiversity Studies and Bioeducation, Banacha 1/3, 90-237, Lodz, Poland*

*^b^Department of Vertebrate Ecology and Zoology, Faculty of Biology, University of Gdansk, Wita Stwosza 59, 80-309 Gdansk, Poland*

*^c^Bird Migration Research Station, Faculty of Biology, University of Gdańsk, Wita Stwosza 59, 80-309, Gdansk, Poland*

*^d^Silesian Ornithological Society, Sienkiewicza 21, 50-335 Wroclaw, Poland*

**corresponding authors:* [*aleksandra.janiszewska2@edu.uni.lodz.pl*](mailto:aleksandra.janiszewska2@edu.uni.lodz.pl)*,* [*maciej.bartos@biol.uni.lodz.pl*](mailto:maciej.bartos@biol.uni.lodz.pl)

**TABLE S1 |** Associations of total louse fly prevalence with host traits among migrating birds (subsampled dataset: n = 105 avian species with at least 10 individuals screened). Coefficient estimates and corresponding 95% credibility limits (CL) were derived from phylogenetically-informed Bayesian mixed models (full and reduced). All categorical variables were coded relative to the following reference levels: Wetland for habitat, Vertivore for trophic niche, and Cavity for nest type. Significant coefficients are marked in bold.

| Predictor | Estimate | Lower 95% CL | Upper 95% CL | p |
| --- | --- | --- | --- | --- |
| *Full model* | | | | |
| Intercept | -1.904 | -6.208 | 2.304 | 0.372 |
| Age (proportion adult birds) | 1.392 | -0.603 | 3.658 | 0.196 |
| Date | 0.005 | -0.006 | 0.017 | 0.382 |
| Log body mass | 0.560 | -0.240 | 1.530 | 0.208 |
| Log clutch size | -0.312 | -1.397 | 0.656 | 0.552 |
| Breeding latitude | 0.015 | -0.017 | 0.048 | 0.354 |
| **Migration distance** | **-0.0001** | **-0.0003** | **0.000** | **0.018** |
| Annual mean temperature | 0.007 | -0.003 | 0.017 | 0.172 |
| **Annual mean precipitation** | **-0.006** | **-0.011** | **-0.002** | **0.006** |
| Habitat: Grassland | -0.946 | -2.596 | 0.490 | 0.216 |
| Habitat: Other | -0.184 | -1.518 | 0.908 | 0.776 |
| Habitat: Shrubland | -0.376 | -1.291 | 0.519 | 0.404 |
| Habitat: Woodland | -0.877 | -1.712 | -0.023 | 0.064 |
| Trophic niche: Aquatic predator | -1.719 | -3.718 | 0.634 | 0.0146 |
| Trophic niche: Herbivore | -1.719 | -3.718 | 0.634 | 0.146 |
| Trophic niche: Invertivore | -1.406 | -3.370 | 0.570 | 0.172 |
| **Trophic niche: Omnivore** | **-1.740** | **-3.224** | **-0.309** | **0.030** |
| Nest: Open elevated | 0.213 | -0.532 | 0.831 | 0.536 |
| Nest: Open ground | 0.129 | -1.114 | 1.403 | 0.856 |
| *Reduced model* |  |  |  |  |
| Intercept | -0.988 | -4.867 | 2.526 | 0.614 |
| Percent of adult birds | 1.583 | -0.601 | 3.650 | 0.152 |
| Log body mass | 0.683 | -0.116 | 1.456 | 0.080 |
| Migration distance | 0.000 | 0.000 | 0.000 | 0.070 |
| Annual mean temperature | 0.004 | -0.004 | 0.014 | 0.322 |
| **Annual mean precipitation** | **-0.006** | **-0.010** | **-0.002** | **0.004** |
| Habitat: Grassland | -0.947 | -2.424 | 0.337 | 0.162 |
| Habitat: Other | -0.454 | -1.528 | 0.619 | 0.412 |
| Habitat: Shrubland | -0.342 | -1.192 | 0.571 | 0.446 |
| **Habitat: Woodland** | **-0.970** | **-1.750** | **-0.103** | **0.028** |
| Trophic niche: Aquatic predator | -1.898 | -3.983 | 0.223 | 0.084 |
| Trophic niche: Herbivore | -1.383 | -3.220 | 0.562 | 0.152 |
| Trophic niche: Invertivore | -1.174 | -2.631 | 0.334 | 0.120 |
| **Trophic niche: Omnivore** | **-1.688** | **-3.382** | **-0.3433** | **0.046** |

**TABLE S2 |** Inter-annual variation in total louse fly prevalence of migrating birds (full dataset: n = 157 avian species). Year was included either as a linear covariate to test for directional changes in prevalence over the sampling period (Model S2.1) or as a fixed factor to test for differences in prevalence among years (Model S2.2). Year 2014 was used as reference level. Coefficient estimates and corresponding 95% credibility limits (CL) were derived from phylogenetically-informed Bayesian mixed models.

| Predictor | Estimate | Lower 95% CL | Upper 95% CL | p |
| --- | --- | --- | --- | --- |
| *Model S2.1* | | | | |
| Intercept | 0.651 | -5.169 | 6.387 | 0.828 |
| Year (linear covariate) | -0.004 | -0.009 | 0.000 | 0.086 |
| *Model S2.2* |  |  |  |  |
| Intercept | -2.881 | -7.322 | 1.774 | 0.202 |
| Year: 2015 | 0.111 | -1.014 | 1.234 | 0.866 |
| Year: 2016 | -0.694 | -2.278 | 0.778 | 0.366 |
| Year: 2017 | -0.423 | -1.624 | 0.671 | 0.484 |
| Year: 2018 | -0.147 | -1.032 | 0.676 | 0.756 |
| Year: 2019 | 0.464 | -0.400 | 1.239 | 0.310 |
| Year: 2020 | 0.436 | -0.393 | 1.271 | 0.354 |
| Year: 2021 | -0.364 | -1.231 | 0.488 | 0.402 |
| Year: 2022 | -0.254 | -1.157 | 0.541 | 0.546 |
| Year: 2023 | -0.694 | -1.589 | 0.199 | 0.138 |

**TABLE S3 |** Associations of identification-adjusted prevalence of Ornithomya avicularia with host traits among migrating birds (full dataset: n = 157 avian species). Coefficient estimates and corresponding 95% credibility limits (CL) were derived from phylogenetically-informed Bayesian mixed models (full and reduced). Significant coefficients are bolded.

| Predictor | Estimate | Lower 95% CL | Upper 95% CL | p |
| --- | --- | --- | --- | --- |
| *Full model* | | | | |
| Intercept | -4.091 | -9.496 | 1.417 | 0.134 |
| Age (proportion adult birds) | -0.528 | -3.092 | 1.854 | 0.710 |
| Date | -0.002 | -0.021 | 0.014 | 0.834 |
| **Log body mass** | **2.400** | **1.101** | **3.534** | **<0.001** |
| Log clutch size | -0.469 | -1.868 | 1.152 | 0.570 |
| Breeding latitude | 0.007 | -0.044 | 0.057 | 0.800 |
| Migration distance | -0.0002 | -0.0004 | 0.0000 | 0.086 |
| Annual mean temperature | 0.004 | -0.010 | 0.019 | 0.562 |
| **Annual mean precipitation** | **-0.006** | **-0.012** | **0.000** | **0.024** |
| Habitat: Grassland | -1.347 | -3.180 | 0.635 | 0.190 |
| Habitat: Other | -0.979 | -3.231 | 1.264 | 0.388 |
| Habitat: Shrubland | -0.713 | -2.412 | 0.817 | 0.350 |
| Habitat: Woodland | -1.097 | -2.323 | 0.364 | 0.114 |
| Trophic niche: Aquatic predator | -1.569 | -4.140 | 1.648 | 0.256 |
| Trophic niche: Herbivore | -1.157 | -3.701 | 1.750 | 0.408 |
| Trophic niche: Invertivore | -0.554 | -2.414 | 1.338 | 0.560 |
| Trophic niche: Omnivore | -0.999 | -2.939 | 0.838 | 0.304 |
| Nest**:** Open elevated | 1.117 | -0.199 | 2.358 | 0.086 |
| Nest: Open ground | 1.337 | -0.657 | 3.159 | 0.174 |
| *Reduced model* | | | | |
| Intercept | -5.882 | -10.270 | -0.876 | 0.028 |
| **Log body mass** | **2.628** | **1.599** | **3.724** | **<0.001** |
| **Migration distance** | **-0.0002** | **-0.0004** | **0.0000** | **0.034** |
| **Annual mean precipitation** | **-0.006** | **-0.010** | **-0.002** | **0.004** |
| Habitat: Grassland | -1.325 | -3.158 | 0.324 | 0.132 |
| Habitat: Other | -0.839 | -2.713 | 1.265 | 0.416 |
| Habitat: Shrubland | -0.588 | -1.729 | 0.639 | 0.332 |
| **Habitat: Woodland** | **-1.200** | **-2.199** | **-0.162** | **0.018** |
| **Nest: Open elevated** | **1.132** | **-0.018** | **2.279** | **0.046** |
| Nest: Open ground | 1.373 | -0.540 | 3.215 | 0.136 |

**TABLE S4 |** Associations of identification-adjusted prevalence of Ornithomya fringillina with host traits among migrating birds (full dataset: n = 157 avian species). Coefficient estimates and corresponding 95% credibility limits (CL) were derived from phylogenetically-informed Bayesian mixed models (full and reduced). Significant coefficients are bolded.

| Predictor | Estimate | Lower 95% CL | Upper 95% CL | p |
| --- | --- | --- | --- | --- |
| *Full model* | | | | |
| Intercept | -1.652 | -6.995 | 3.501 | 0.538 |
| Age (proportion adult birds) | -0.151 | -3.021 | 2.199 | 0.952 |
| Date | 0.006 | -0.009 | 0.024 | 0.456 |
| **Log body mass** | **-2.466** | **-4.006** | **-0.848** | **0.002** |
| Log clutch size | -0.494 | -2.052 | 0.928 | 0.562 |
| Breeding latitude | 0.016 | -0.023 | 0.051 | 0.412 |
| Migration distance | -0.0002 | -0.0003 | 0.0000 | 0.058 |
| Annual mean temperature | 0.008 | -0.005 | 0.022 | 0.224 |
| Annual mean precipitation | -0.005 | -0.011 | 0.000 | 0.074 |
| Habitat: Grassland | 0.206 | -2.158 | 2.235 | 0.860 |
| **Habitat: Other** | **-2.523** | **-5.099** | **-0.060** | **0.036** |
| Habitat: Shrubland | -0.193 | -1.301 | 0.904 | 0.750 |
| **Habitat: Woodland** | **-1.659** | **-2.680** | **-0.382** | **0.010** |
| Trophic niche: Aquatic predator | -3.036 | -7.051 | 0.739 | 0.146 |
| Trophic niche: Herbivore | 1.366 | -1.467 | 4.179 | 0.352 |
| Trophic niche: Invertivore | 1.229 | -1.375 | 3.873 | 0.348 |
| Trophic niche: Omnivore | 1.177 | -1.402 | 3.731 | 0.380 |
| Nest**:** Open elevated | 0.250 | -0.678 | 1.192 | 0.562 |
| Nest: Open ground | -0.707 | -2.612 | 1.170 | 0.454 |
| *Reduced model* |  |  |  |  |
| Intercept | -0.832 | -5.301 | 3.904 | 0.752 |
| **Log body mass** | **-2.296** | **-3.720** | **-0.909** | **<0.001** |
| Migration distance | -0.0001 | -0.0003 | 0.0000 | 0.132 |
| Annual mean temperature | 0.005 | -0.005 | 0.017 | 0.330 |
| Annual mean precipitation | -0.005 | -0.009 | 0.001 | 0.080 |
| Habitat: Grassland | -0.500 | -2.336 | 0.942 | 0.534 |
| **Habitat: Other** | **-2.931** | **-5.262** | **-0.906** | **0.006** |
| Habitat: Shrubland | -0.359 | -1.438 | 0.695 | 0.502 |
| **Habitat: Woodland** | **-1.788** | **-2.811** | **-0.668** | **0.002** |
| Trophic niche: Aquatic predator | -2.907 | -7.657 | 1.871 | 0.202 |
| Trophic niche: Herbivore | 1.832 | -0.964 | 4.665 | 0.180 |
| Trophic niche: Invertivore | 1.362 | -1.234 | 4.078 | 0.328 |
| Trophic niche: Omnivore | 1.486 | -1.016 | 4.275 | 0.288 |

< # >

**TABLE S5 |** Associations of Ornithomya avicularia prevalence with host traits among migrating birds (subsampled dataset: n = 105 avian species with at least 10 individuals screened). Coefficient estimates and corresponding 95% credibility limits (CL) were derived from phylogenetically-informed Bayesian mixed models (full and reduced). All categorical variables were coded relative to the following reference levels: Wetland for habitat, Vertivore for trophic niche, and Cavity for nest type. Significant coefficients are marked in bold.

| Predictor | Estimate | Lower 95% CL | Upper 95% CL | p |
| --- | --- | --- | --- | --- |
| *Full model* | | | | |
| Intercept | -3.204 | -8.621 | 2.719 | 0.274 |
| Age (proportion adult birds) | 1.867 | -1.724 | 5.643 | 0.328 |
| Date | -0.005 | -0.026 | 0.014 | 0.622 |
| **Log body mass** | **2.720** | **1.420** | **4.036** | **<0.001** |
| Log clutch size | -0.510 | -2.071 | 1.243 | 0.512 |
| Breeding latitude | 0.003 | -0.057 | 0.077 | 0.934 |
| Migration distance | -0.0002 | -0.0005 | 0.00005 | 0.128 |
| Annual mean temperature | 0.007 | -0.010 | 0.029 | 0.476 |
| **Annual mean precipitation** | **-0.008** | **-0.016** | **0.00003** | **0.032** |
| Habitat: Grassland | -0.713 | -3.136 | 1.484 | 0.552 |
| Habitat: Other | -0.136 | -2.356 | 2.146 | 0.964 |
| Habitat: Shrubland | -0.248 | -1.942 | 1.669 | 0.798 |
| Habitat: Woodland | -0.848 | -2.570 | 0.680 | 0.288 |
| Trophic niche: Aquatic predator | -1.517 | -4.555 | 1.154 | 0.296 |
| Trophic niche: Herbivore | -1.669 | -4.268 | 1.369 | 0.274 |
| Trophic niche: Invertivore | -0.633 | -2.722 | 1.313 | 0.520 |
| Trophic niche: Omnivore | -1.141 | -3.140 | 1.005 | 0.258 |
| Nest: Open elevated | 0.993 | -0.366 | 2.361 | 0.150 |
| Nest: Open ground | 1.131 | -0.877 | 3.646 | 0.322 |
| *Reduced model* |  |  |  |  |
| Intercept | -5.282 | -9.541 | -0.340 | 0.036 |
| **Log body mass** | **2.794** | **1.807** | **4.008** | **<0.001** |
| Migration distance | -0.0002 | -0.0003 | 0.00001 | 0.052 |
| **Annual mean precipitation** | **-0.008** | **-0.013** | **-0.004** | **0.002** |
| **Nest: Open elevated** | **1.187** | **0.000** | **2.350** | **0.046** |
| Nest: Open ground | 1.336 | -0.505 | 3.130 | 0.142 |

**TABLE S6 |** Associations of Ornithomya fringillina prevalence with host traits among migrating birds (subsampled dataset: n = 105 avian species with at least 10 individuals screened). Coefficient estimates and corresponding 95% credibility limits (CL) were derived from phylogenetically-informed Bayesian mixed models (full and reduced). All categorical variables were coded relative to the following reference levels: Wetland for habitat, Vertivore for trophic niche, and Cavity for nest type. Significant coefficients are marked in bold.

| Predictor | Estimate | Lower 95% CI | Upper 95% CI | p |
| --- | --- | --- | --- | --- |
| *Full model* | | | | |
| Intercept | -0.930 | -6.058 | 3.904 | 0.724 |
| Age (proportion adult birds) | 0.508 | -2.896 | 3.510 | 0.710 |
| Date | 0.001 | -0.013 | 0.018 | 0.910 |
| **Log body mass** | **-2.314** | **-3.757** | **-0.751** | **0.002** |
| Log clutch size | -0.716 | -2.254 | 0.965 | 0.380 |
| Breeding latitude | 0.026 | -0.011 | 0.065 | 0.174 |
| **Migration distance** | **-0.0002** | **-0.0004** | **-0.00005** | **0.020** |
| Annual mean temperature | 0.009 | -0.006 | 0.023 | 0.220 |
| Annual mean precipitation | -0.005 | -0.011 | 0.001 | 0.120 |
| Habitat: Grassland | 0.525 | -1.881 | 3.001 | 0.684 |
| Habitat: Other | -2.228 | -4.977 | -0.096 | 0.062 |
| Habitat: Shrubland | -0.191 | -1.428 | 0.965 | 0.698 |
| **Habitat: Woodland** | **-1.774** | **-3.046** | **-0.560** | **0.006** |
| Trophic niche: Aquatic predator | -3.067 | -7.845 | 1.145 | 0.158 |
| Trophic niche: Herbivore | 1.200 | -1.825 | 4.063 | 0.424 |
| Trophic niche: Invertivore | 1.185 | -1.827 | 3.920 | 0.394 |
| Trophic niche: Omnivore | 1.058 | -1.835 | 3.932 | 0.462 |
| Nest: Open elevated | 0.136 | -0.795 | 1.051 | 0.796 |
| Nest: Open ground | -0.504 | -2.467 | 1.427 | 0.616 |
| *Reduced model* |  |  |  |  |
| Intercept | -1.337 | -5.881 | 2.704 | 0.554 |
| **Log body mass** | **-2.116** | **-3.555** | **-0.896** | **<0.001** |
| Breeding latitude | 0.027 | -0.012 | 0.056 | 0.114 |
| **Migration distance** | **-0.0002** | **-0.0003** | **-0.00003** | **0.026** |
| Annual mean temperature | 0.011 | -0.001 | 0.024 | 0.092 |
| **Annual mean precipitation** | **-0.006** | **-0.012** | **0.000** | **0.044** |
| Habitat: Grassland | -0.070 | -1.837 | 1.647 | 0.964 |
| **Habitat: Other** | **-2.365** | **-4.581** | **0.038** | **0.030** |
| Habiat: Shrubland | -0.265 | -1.352 | 0.800 | 0.640 |
| **Habitat: Woodland** | **-1.802** | **-2.925** | **-0.522** | **0.010** |
| Trophic niche: Aquatic predator | -3.540 | -8.282 | 0.943 | 0.120 |
| Trophic niche: Herbivore | 1.291 | -1.850 | 4.250 | 0.388 |
| Trophic niche: Invertivore | 0.897 | -1.686 | 4.000 | 0.526 |
